# Physical Prehabilitation for Older Patients with Cancer before Complex Medical-Surgical Interventions: An Umbrella Review

**DOI:** 10.14336/AD.2024.0957

**Published:** 2025-11-04

**Authors:** Dana Loudovici-Krug, Louise André, Heiner Paul Blumensaat, Marion Granger, Laetitia Stefani, Josephine Kühnemund, Christina Lemhöfer, Claire Falandry

**Affiliations:** ^1^Institute for Physical and Rehabilitation Medicine, Jena University Hospital, Jena, Germany.; ^2^Service de Gériatrie, Hôpital Lyon-Sud, Hospices Civils de Lyon, Pierre-Bénite, France.; ^3^Service de Gériatrie, Hôpital Nord-Ouest, Villefranche sur Saône, France.; ^4^Service de Gériatrie, Centre Hospitalier Métropole Savoie, Chambéry, France.; ^5^Centre Hospitalier Annecy Genevois, 1 Avenue de l’Hôpital, BP 90074, 74374 Pringy, France.; ^6^Service de Gériatrie, Hôpital Lyon-Sud, Hospices Civils de Lyon, Pierre Benite, France.; ^7^CarMeN Laboratory, Inserm U1060, INRA U1397, Université Claude Bernard Lyon 1, INSA Lyon, Université Charles Mérieux, Oullins, France.

**Keywords:** pre-habilitation, elderly, oncogeriatrics, exercise intervention, surgery

## Abstract

Prehabilitation has become a field of increasing interest over recent decades. However, few studies specifically investigated prehabilitation for older patients with cancer. The objective of this umbrella review was to summarize evidence on prehabilitation programs to identify the physical interventions that may be applied with benefit to older cancer patients who will undergo complex medical-surgical procedures. The protocol was registered in Prospero. Major databases, namely PubMed, Embase, CINAHL, Cochrane, Web of Science and Prospero, were searched until summer 2020 and a second search was performed until November 2023. All systematic reviews and meta-analyses were included, dealing with the major topic of prehabilitation for older patients with cancer diagnosis. Among 1425 records (633 until 2020, 792 until November 2023), 14 reviews were selected for inclusion. According to the AMSTAR-2 checklist, the median quality score was 11 (range: 5-12). Total duration of prehabilitation ranged from 1 to 5 weeks, session duration from 20 to 50 minutes, session frequency from 3 to 6 per week. Reported program modes were aerobic and resistance exercises. Concerning the outcome measures, the functional as well as the respiratory status was significantly affected. Quality of life did not benefit significantly, but showed a positive trend. The length of hospital stay was not significantly improved in the majority of the studies. In contrast, most systematic reviews reported significantly lower numbers of total postoperative complications. Functional recovery was enhanced in half of the found reviews. Prehabilitation is a growing field, notably also in reviews focussing on oncological care for elderly patients included in this umbrella review. Aerobic and resistance exercises are the core of the majority of the programs evaluated but their characteristics (total duration, frequency) are partly heterogeneous. Prehabilitation for older patients may also include other modalities of geriatric interventions like nutritional or psychological optimization.

## INTRODUCTION

More than half of all cancers are diagnosed in patients aged over 65 years [1]. Older patients or those with many comorbidities are at high risk of deconditioning shortly after the onset of cancer treatment, especially when this implies major surgery [2]. Prehabilitation aims to enhance the functional capacity before surgery or other major medical interventions in order to decrease morbidity afterwards [2]. Postoperative geriatric events are frequent among patients who undergo cancer surgery [1], therefore the older patients are likely to benefit from prehabilitation strategies. Hence, prehabilitation has become a field of exponentially increasing interest over recent decades [3] and the impact should be illustrated in particular for older patients with cancer. Prehabilitation is announced as “gateway to improving functional outcomes throughout the cancer continuum” [4]. The most common components are exercise, nutrition and psychological management. However, the multimodal list can be supplemented by other aspects such as sleep optimization and cognitive or educational modules [5].

In this context, this umbrella review aims to show all the prehabilitation programs reported in the literature in order to identify the physical prehabilitation interventions available that are applicable to older cancer patients who will undergo complex medical-surgical procedures [6].

## METHODS

The present umbrella review report followed the Preferred Reporting items for Systematic Reviews and Meta-Analyses (PRISMA) statement. Its protocol was registered in PROSPERO (CRD42020100110) and published before conducting the research [7].

Major databases like PubMed (MEDLINE), Embase, CINAHL, Cochrane Library, Web of Science and Prospero were searched in two steps: (1) until summer 2020 and (2) until November 2023. Additional references identified by the authors, for example further citations in the retrieved reviews, were added if they met the inclusion criteria of the umbrella review. Eligible publication types were systematic reviews and meta-analyses.

Due to the expansion of the literature between the first and the second steps, the primary inclusion criteria as defined in the published protocol [7] were tightened during the second one in order to exclude all systematic analyses focused on prehabilitation in patients under 65 years of age or in settings other than cancer.

The search strategy was carried out with a combination of the following keywords: prehabilitation, pre-surgical, preoperative exercise AND aged, elderly AND cancer, oncological AND systematic review OR meta-analysis. Regarding the patient population there were four inclusion criteria: (1) Reviews were eligible if the mean or median age of all patients in included studies combined was 65 and above OR if more than half of the included studies had a mean or median age higher than 65. (2) Furthermore, to be included, the intervention groups had to undergo major oncological surgery and/or complex medical interventions at some point. (3) The majority of the included studies had to be cancer patients only. (4) Finally, there had to be a physical prehabilitation program of any kind.

**Figure 1.**
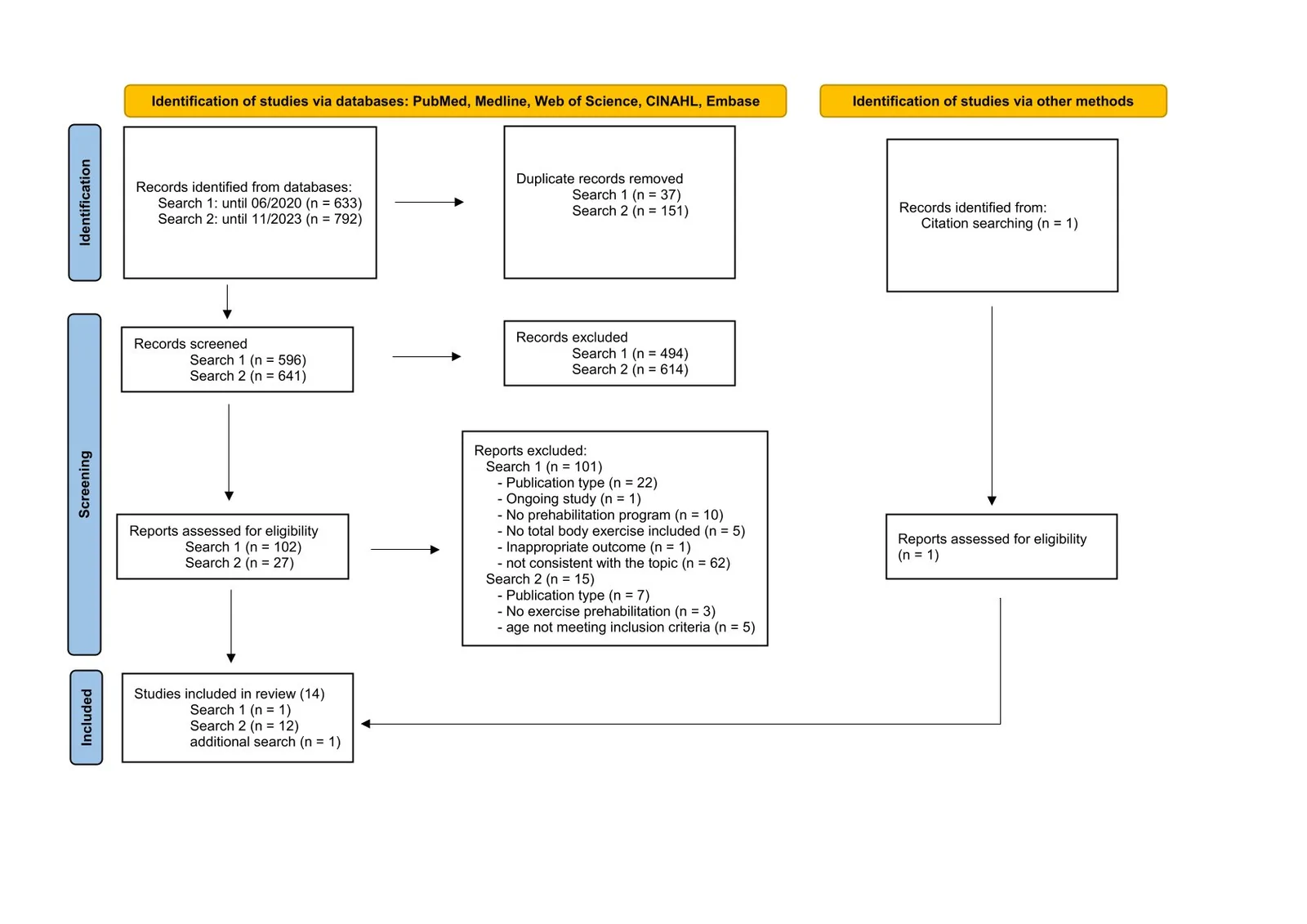
PRISMA Flow Diagram.

Reviews were excluded if the full text was not available, if it was written in any language other than English or if they focused on nutritional, pharmacological, mental, psychiatric or palliative care only. The search as well as the selection of the studies to be included was carried out independently by two reviewers. Any disagreement between reviewers was resolved through discussion and a final agreement was reached.

For each review, the most important study data were extracted: author, publication year, prehabilitation context, number of included studies, number of studies including cancer patients only, total sample size, distribution of gender, characteristics of the prehabilitation programs and the concerning outcomes as well as the effect of prehabilitation on these. The methodological quality of included studies was evaluated using the updated AMSTAR-2 measurement tool [8].

## RESULTS

The database search of both searches provided 1425 records in total (Fig. 1). After removal of 188 duplicates, 1237 records were screened for eligibility: 129 appeared to fulfil the inclusion criteria. Further 116 records had to be excluded because of publication type, missing physical prehabilitation program, inappropriate outcome, lack of consistency with the topic or an unsuitable age range. One eligible study was found via the search of reference lists. A total of 14 reviews [9-22] were included in this umbrella review.

**Table 1:** Assessing the methodological quality of systematic reviews-2 (AMSTAR-2).

	Q1	Q2	Q3	Q4	Q5	Q6	Q7	Q8	Q9	Q10	Q11	Q12	Q13	Q14	Q15	Q16	no meta-analysis		with meta-analysis	
																	critical domains (5)	in total (13)	critical domains (7)	in total (16)
Clifford et al. 2023	+	+	-	pt. +	-	+	-	pt. +	+	-	+	-	+	+	+	+			6	11
Falz et al. 2022	+	+	-	pt. +	+	+	-	pt. +	+	-	+	+	-	+	-	+			4	11
Guo et al. 2022	+	+	-	pt. +	+	+	-	+	+	-	+	+	+	+	-	+			5	12
Jain et al. 2022	+	+	+	pt. +	-	+	-	+	+	-	+	-	+	+	+	+			6	12
Liu et al. 2022	+	pt. +	-	pt. +	+	+	-	pt. +	+	-	+	+	+	+	-	+			5	12
Looijard et al. 2018	+	-	-	pt. +	+	-	-	pt. +	pt. +	-	NO	NO	+	+	NO	-	3	7		
Michael et al. 2021	+	pt. +	+	pt. +	+	-	-	+	pt. +	-	+	+	+	+	-	+			5	12
Pang et al. 2022	+	+	-	pt. +	+	+	-	+	+	-	+	-	+	+	+	+			6	12
Piraux et al. 2021	+	pt. +	-	pt. +	+	+	-	+	+	-	NO	NO	-	+	NO	+	3	9		
Rosero et al. 2019	+	-	-	pt. +	+	+:	pt. +	pt. +	pt. +	-	+	+	+	+	-	+			5	12
Santek et al. 2021	+	-	-	-	+	+	-	pt. +	+	-	NO	NO	-	-	NO	-	1	5		
Thillainadesan et al. 2020	+	+	-	pt. +	+	+	-	pt. +	+	-	+	+	+	+	-	-			5	11
Voorn et al. 2023	+	+	+	pt. +	+	-	-	+	+	-	+	+	-	+	-	+			4	11
Zhang et al. 2022	+	+	-	pt. +	-	+	-	pt. +	+	-	+	+	-	+	+	+			5	11

NO = no meta-analysis, pt. = partial, Q = question, + = yes, - = no

**Table 2:** Characteristics of included studies.

Author	Context	Nb of included studies (Nb of studies including cancer patients only)	total sample size	female / male in %	Aerobic / Resistance (Nb of studies)	Median session length in minutes (range)	Median Nb of sessions per week (range)	Median total duration in weeks (range)	Additional prehabilitation modalities
				NR - not reported					1-nutritional/ 2-respiratory/ 3-psychological
**Clifford et al. 2023**	liver, lung, colorectal, urologic and mixed major abdominal surgeries (mainly cancer)	10 (8)	832	29/71	10/5	30 (18-60)	3 (2-5)	4 (3-8)	1, 2
**Falz et al. 2022**	colon and rectal cancer	23 (23)	1461	NR	23/7	32.5 (15-60)	5.5 (2-7)	4 (2-14)	1, 2, 3, educational
**Guo et al. 2022**	colorectal, esophageal, gastrointestinal or mixed cancer	9 (9)	1313	NR	4/4	37.5 (30-45)	3 (2-3)	3 (2-4)	1, 3
**Jain et al. 2022**	colorectal, hepatopancreatobiliary, urological and general abdominal surgery	25 (16)	4210	NR	17/13	47.5 (25-60)	3 (1-5)	4 (4)	1, 3
**Liu et al. 2022**	colorectal and lung cancer; liver resection	10 (9)	1553	NR	7/8	NR	NR	4 (2-6)	1, 3
**Michael et al. 2021**	lung, colorectal, rectal, esophagogastric, liver and prostate cancer	23 (23)	1223	NR	13/11	50 (30-210)	3.3 (2-10)	4 (1-16)	none
**Pang et al. 2022**	colorectal, oesophagogastric, bladder cancer, cancer of the peri-ampullary complex; major digestive tract diseases	9 (8)	705	NR	NR	NR	NR	4 (2.5-6)	1, 3
**Piraux et al. 2021**	esophagogastric cancer	7 (7)	645	22/78	4/4	50 (20-75)	5 (2-7)	4 (1-19)	2
**Rosero et al. 2019**	non-small-cell lung cancer, chronic obstructive pulmonary disease, respiratory diseases	10 (10)	676	40/60	9/3	20 (20-60)	6 (3-7)	3 (1-4)	2
**Santek & Kirac 2021**	major abdominal, colorectal, rectal, liver surgery	10 (10)	1058	36/64	NR	NR	NR	NR (2-6)	none
**Thillainadesan et al. 2020**	urologic, gastrointestinal or mixed cancers; vascular, trauma, cardiothoracic, colorectal, general surgery	24 (15)	3026	48/52	NR	NR	NR	NR (2-6)	multicomponent geriatric programs, cognitive training
**Voorn et al. 2023**	non-small-cell lung cancer	16 (16)	2094	NR	15/9	30 (15-120)	5 (1-7)	1 (1-4)	2
**Zhang et al. 2022**	colorectal surgery (mainly cancer)	15 (10)	1306	NR	NR	NR	NR	NR	1, 3
**Looijaard et al. 2018**	colorectal cancer surgery	6 (6)	516	NR	3/3	50 (30-60)	3 (2-3)	5 (4-5)	1, 3

### Quality of reviews

The 14 involved reviews included a total of 116 studies (with duplications 197) with 13.025 patients (with duplications 20.618). The most frequent cancer sites were abdominal (examined in 85.7 % of included reviews), concerning the lung (examined in 35.7 % of included reviews), and urological (examined in 35.7 % of included reviews). The quality of the records, evaluated by using the AMSTAR-2 checklist (Table 1), is heterogeneous. The systematic reviews with meta-analysis, reached four to six points of the seven critical domains and 11 to 12 points of 16 points in total. The mostly missing critical domain refers to the lack of a list of excluded studies and their justifications summarized in question seven. Moreover, the third question (concerning the selection of study designs), question 10 (concerning the sources of funding for the studies included in the review) and question 15 (concerning the assessment of publication biases) had to be mainly assessed as negative.

### Review characteristics

The Table 2 shows several characteristics of the included reviews. Median duration of prehabilitation ranged from 1 to 5 weeks (median: 4 weeks), session duration ranged from 20 to 50 minutes (median: 37.5 minutes), session frequency ranged from 3 to 6 per week (median: 3.3 per week). Considering the type of intervention, two modes of exercise were reported primarily: aerobic and resistance exercises. The first one included any type of endurance activity, that increases the heartrate over a prolonged period of time, and the second one referred to any kind of weight and strength training, respectively.

In addition to total body physical prehabilitation, other types of prehabilitation were reported in 12 reviews (85.7 %), most frequently nutritional (n = 8; 57.1 %) and psychological (n = 7; 50 %), followed by respiratory programs (n = 5; 35.7 %). Regarding the characteristics of the studied population information provided in the reviews was sparce. Apart from the age of the participants, some reviews reported the gender distribution (Table 2). No other patient characteristics were mentioned.

The adherence, meaning the completion of the scheduled intervention, was reported in 4 of the 14 reviews [10, 13, 17, 20]. In these reviews median adherence ranged from 78 to 93.5 percent. Overall, continuous supervised in-hospital or outpatient clinic interventions had the highest adherence and unsupervised home-based interventions the lowest [10-13]. Although measures to increase adherence are named in the methodology, there are ultimately no statements on the compliance rate [15].

**Table 3:** Summary of outcomes assessed in included reviews.

time of assessment pre medical-surgical procedure (post exercise prehabilitation)	outcome parameter	authors	reported quality			significance of positive effect			Nb of included studies (Nb of patients)
			low	moderate	high	significant	tendency	no difference/ ° nb	
	6MWT	Clifford et al. 2023	x			↑			5 (584)
		**Falz et al. 2022**		x		↑			16 (2298)
		**Jain et al. 2022**				↑			10 (885)
		**Pang et al. 2022**	x			↑			7 (645)
		**Rosero et al. 2019**	x			↑			6 (359)
	Quality of life (SF-36 questionaire)	Clifford et al. 2023	x				↗		4 (214)
		**Jain et al. 2022**						↔	6 (618)
		**Looijaard et al. 2018**						↔	2 (164)
		**Pang et al. 2022**					↗		5 (585)
		**Santek & Kirac 2021**					↗		NR
	Nb of adverse events (Nb of Participants)	Clifford et al. 2023						° 1	10 (786)
		**Falz et al. 2022**						° 5	8 (1072)
		**Piraux et al. 2021**						° 0	4 (356)
		**Thillainadesan et al. 2020**						° 0	8 (1466)
	VO2peak	Clifford et al. 2023		x		↑			8 (627)
		**Rosero et al. 2019**	x			↑			3 (368)
		**Santek & Kirac 2021**					↗		NR
	mental component summary score	Clifford et al. 2023	x					↔	3 (191)
		**Jain et al. 2022**					↗		6 (618)
	maximal inspiratory pressure	Piraux et al. 2021				↑			3 (263)
		**Santek & Kirac 2021**				↗			NR
	inspiratory muscle endurance	Piraux et al. 2021				↑			2 (224)
		**Santek & Kirac 2021**				↗			NR
	**peak power output (cardiopulmonary test)**	Clifford et al. 2023	x			↑			6 (336)
	**anaerobic threshold**	Clifford et al. 2023	x			↑			7 (587)
	**dyspnoea**	Rosero et al. 2019				↑			4 (234)
time of assessment post medico-surgical procedure	length of hospital stay	Clifford et al. 2023	x					↔	8 (729)
		**Falz et al. 2022**						↔	12 (1418)
		**Guo et al. 2022**				↑			7 (859)
		**Jain et al. 2022**						↔	NR (3761)
		**Liu et al. 2022**						↔	9 (1190)
		**Looijaard et al. 2018**						↔	3 (202)
		**Pang et al. 2022**	x					↔	9 (705)
		**Santek & Kirac 2021**					↗		NR
		**Thillainadesan et al. 2020**				↑			17 (2600)
		**Voorn et al. 2023**	x			↑			10 (1661)
		**Zhang et al. 2022**						↔	9 (655)
		**Piraux et al. 2021**						↔	6 (455)
		**Rosero et al. 2019**	x			↑			6 (509)
	total postoperative complications	Clifford et al. 2023		x		↑			8 (770)
		**Falz et al. 2022**					↗		11 (729)
		**Guo et al. 2022**				↑			7 (858)
		**Jain et al. 2022**				↑			19 (3180)
		**Liu et al. 2022**						↔	10 (1553)
		**Looijaard et al. 2018**						↔	3 (202)
		**Pang et al. 2022**		x		↑			9 (705)
		**Thillainadesan et al. 2020**				↑			13 (1833)
		**Voorn et al. 2023**	x			↑			15 (2009)
		**Zhang et al. 2022**						↔	14 (1265)
		**Piraux et al. 2021**						↔	5 (395)
		**Rosero et al. 2019**	x			↑			8 (605)
	functional recovery via 6MWT 4-8 wks post	Guo et al. 2022					↗		2 (240)
		**Jain et al. 2022**						↔	NR (649)
		**Liu et al. 2022**				↑			3 (260)
		**Liu et al. 2022**						↔	3 (227)
		**Looijaard et al. 2018**				↑			2 (164)
		**Michael et al. 2021**				↑			11 (820)
		**Pang et al. 2022**	x			↑			4 (278)
		**Zhang et al. 2022**						↔	3 (299)
	severe postoperative complications	Guo et al. 2022				↑			5 (505)
		**Jain et al. 2022**						↔	NR (681)
		**Pang et al. 2022**		x				↔	6 (428)
		**Voorn et al. 2023**		x		↑			7 (1378)
	30-day mortality	Guo et al. 2022					↗		4 (439)
		**Liu et al. 2022**						↔	4 (1062)
		**Voorn et al. 2023**	x					↔	6 (1200)
		**Piraux et al. 2021**						↔	2 (224)
	Hospital readmission within 30 days	Guo et al. 2022						↔	5 (431)
		**Jain et al. 2022**						↔	NR (2518)
		**Liu et al. 2022**						↔	7 (1224)
		**Pang et al. 2022**		x				↔	6 (536)
	3-month mortality	Guo et al. 2022						↔	2 (192)
		**Jain et al. 2022**						↔	19 (3315)
	postoperative pulmonary complications	Rosero et al. 2019				↑			6 (519)
		**Voorn et al. 2023**		x		↑			10 (1558)
	**physical component summary (12 weeks post)**	Clifford et al. 2023						↔	2 (103)
	**Hospital readmission within 3 months**	Guo et al. 2022						↔	2 (291)
	**Emergency department visit within 30 days**	Pang et al. 2022			x			↔	5 (496)

NR = not reported, ↑ = significant and positive effect, ↗ = no significant effect, positive trend; ↔ = no effect found, Nb = number

### Outcomes

In total 21 different outcome measures were reported in the included reviews (Table 3). They were further divided into two subgroups: *post exercise prehabilitation* (n=10) and *post medical-surgical procedure* (n=11). The deciding factor for classification was the time of the outcome measurement, which was after the prehabilitation phase but before the medical-surgical procedure in some cases and after the medical-surgical procedure in others. The most common ones in the first subgroup were the 6-minute-walk-test (6MWT), quality of life (including mental component summary score) and the number of adverse events, additionally the volume of oxygen peak, the peak power output, maximum inspiratory pressure, dyspnoea, inspiratory muscle endurance and anaerobic threshold.

In the second subgroup, respectively, the most common measured outcomes were the length of hospital stay, total postoperative complications and functional recovery or the physical component summary scale. Moreover, severe just as pulmonary complications were regarded, as well as the 30-day mortality, 3-month mortality, hospital readmission within 30 days or 3 months and the emergency department visit within 30 days of operation.

Concerning the 6MWT all five reporting reviews found a significant and positive effect of exercise prehabilitation [9, 10, 15, 17, 22]. The respiratory status concerning oxygen peak, inspiratory pressure and muscle endurance, as well as anaerobic threshold or dyspnoea, is influenced positively and even significant for the most part [9, 17, 18, 16]. Quality of life did not benefit significantly in any of the five reporting reviews, however there was a positive trend in three of them [9, 15, 18, 13, 22]. The number of adverse events was zero in two of four reporting reviews [16, 19] and respectively one and five in the other two [9, 10].

The length of hospital stay, reported in 13 reviews, was found to be not or not significantly improved in nine reviews [9, 10, 12, 13, 15, 18, 21, 16, 22] and to be improved significantly in four reviews [11, 19, 20, 17]. The total postoperative complications were found to be significantly lower in seven of 12 reviews [9, 11, 15, 19, 20, 17, 22] and not improved in five [10, 12, 13, 16, 21]. Functional recovery, which was measured by means of the 6MWT, four to eight weeks after the medical-surgical intervention, was reported in seven reviews. In one of these studies, the fourth- and eighth-week post condition were reported separately of one another so there are eight results to be evaluated in total. Four of those found a significantly better recovery in the intervention group [12–;15]. No statistically significant difference was found in four studies [12, 21, 22], of which one found at least a positive trend in the intervention group [11].

## DISCUSSION

This umbrella review illustrates that prehabilitation, especially for older patients with cancer, is a field of interest that deserves more attention in the future than it does today. The effects are diverse and partly complementary. Nevertheless, there is strong evidence of positive effects on outcome measures, such as functional capacity and recovery as well as postoperative complications. Some positive effects were also observed on the length of hospital stay. The influence on quality of life, hospital readmission and mortality, however, require further research, since no significant effects of prehabilitation could be determined.

The length, intensity and frequency of exercise sessions, seem to be different between older patients and the general population. These parameters should be adopted to prehabilitation for older patients. For instance, Fell and Williams [23] demonstrated that alternating training and recovery phases allowing supercompensation lead to improved performance in older patients. This should be considered as recovery is delayed due to increased exercise-induced fatigue and impaired adaptation after exercise. The delayed recovery is heterogeneous and varies according to the training impulse and the initial level of performance [23]. Furthermore, Cadore et al. [24] reported that the intensity of exercise is important as an increase of intensity can induce functional status improvement and decrease falls, even in a frail nonagenarian population. However, when the intensity of training impulse increases, the fatigue phase is more intense, and the recovery takes longer. This may be the consequence of the atrophy of muscle expressing the fast myosin heavy chain isoform, observed in the elderly population [24]. This highlights the necessity of adjusting these parameters to older patients' functional status, to supervise exercises and modulate the program according to the tolerance reported by the patients. Home sessions in combination with tele-supervision could facilitate the adherence to the program. Innovative ways to allow the supervision of these sessions at home are being developed, such as connected electronic tools, remote monitoring of heart rate or vital parameters, or the use of logbooks to monitor program adherence [25].

This umbrella review focused on physical prehabilitation programs, but additional prehabilitation modalities were also reported. More than half of the reviews reported nutritional care in their prehabilitation program, which seems to be particularly important for the older population as loss of muscle mass with aging is primarily due to decreased muscle protein synthesis, mainly caused by insufficient protein intake [26]. Other additional interventions, tailored for an older population, may improve patient outcomes, such as health education, psychological care or adaptation to the patient's cognitive status. It is interesting to notice that among the comments made on the paper authored by Carli et al. [27], Kako et al. proposed that cognitive impairment could have affected the ability of patients to participate in the prehabilitation program [28]. The last point of note is that outcome measures differ from one review to another. The most frequently used was postoperative complications, usually evaluated using Clavien-Dindo classification [17, 16, 10, 15, 20, 11, 22]. This measurement of direct surgical complications is of importance, but may not be suitable for older people who undergo major oncological surgery. In fact, this population is at high risk of hospital readmission in the 30 days following surgery mainly due to geriatrics events, when are not accounted by Clavien-Dindo classification [1]. It is therefore critical to include geriatric events, but also quality of life, or functional status that are more pertinent than mortality. Surgery on older patients contains higher risks compared to the average population. Therefore, if a procedure promises positive long-term effects, is a crucial information. Several parameters recording effects exceeding four weeks were included in some of the reviews. Of those, functional recovery four to eight weeks post-surgery was the only one found to be positively influenced by prehabilitation programs [12-15], whereas the hospital readmission and emergency department visits do not seem to be influenced [11, 12, 15, 22].

**Table 4:** Overlapping of the referring studies in the included systematic reviews.

	review authors	Clifford et al. 2023	Falz et al. 2022	Guo et al. 2022	Jain et al. 2022	Liu et al. 2022	Looijaard et al. 2018	Michael et al. 2021	Pang et al. 2022	Piraux et al. 2021	Rosero et al. 2019	Santek & Kirac 2021	Thillainadesan et al. 2020	Voorn et al. 2023	Zhang et al. 2022
study authors	number of studies included in the review (in total) →	12	23	9	25	10	6	21	9	7	10	10	24	16	15
	**number of how often study is used in the reviews (min 2x) ↓**														
**Gillis et al. 2014**	8		x		x	x	x	x	x				x		x
**Carli et al. 2020**	7		x	x	x	x		x	x						x
**Barberan-Garcia et al. 2018**	6	x	x		x				x			x	x		
**Bousquet-Dion et al. 2018**	6		x		x	x		x	x						x
**Chia et al. 2016**	6		x	x	x	x							x		x
**Li et al. 2013**	6				x	x	x	x					x		x
**Fulop et al. 2021**	5		x		x	x			x						x
**van Rooijen et al. 2019**	5	x	x		x	x									x
**Berkel et al. 2022**	4	x	x	x											x
**Dronkers et al. 2010**	4		x				x					x	x		
**López-Rodríguez-Arias et al. 2021**	4		x		x				x						x
**Benzo et al. 2011**	3							x			x			x	
**Dunne et al. 2016**	3	x						x				x			
**Lai, Huang et al. 2017**	3							x			x			x	
**Licker et al. 2017**	3	x									x			x	
**West et al. 2015**	3	x	x					x							
**Ausania et al. 2019**	2				x				x						
**Banerjee et al. 2018**	2	x											x		
**Burden et al. 2011**	2						x								x
**Carli et al. 2010**	2		x									x			
**Chen et al. 2017**	2							x					x		
**Hempenius et al. 2013**	2			x									x		
**Huang et al. 2017**	2										x			x	
**Janssen et al. 2019**	2		x		x										
**Jensen, Peterson et al. 2014**	2				x								x		
**Karenovics et al. 2017**	2							x			x				
**Karlsson et al. 2019**	2		x									x			
**Lai et al. 2016**	2										x			x	
**Minnella et al. 2018**	2							x	x						
**Minnella et al. 2020**	2		x		x										
**Mora López et al. 2020**	2		x		x										
**Northgraves et al. 2020**	2		x												x
**Ommundsen et al. 2018**	2			x									x		
**Sebio García et al. 2016**	2	x												x	
**Sebio García et al. 2017**	2							x			x				
**Stefanelli et al. 2013**	2	x									x				
**Wang et al. 2020**	2				x	x									

Some parameters of prehabilitation programs are not described in the reviews included in this umbrella review but they seem to be important to take into consideration. For instance, it was not possible to analyze the strength, velocity and power of exercises. Yet, Kostka et al. demonstrated that in a frail population the change in functional status correlated positively with strength but not with velocity or power [29]. Another interesting point not reported in this umbrella review is the need to prevent overtraining syndrome during a prehabilitation program. Symptoms of overtraining (such as muscle weakness, chronic fatigue, reduced motivation, underperformance, decrease in exercise capacity/intensity, loss of appetite, and weight loss) become more frequent with age and are linked to a greater susceptibility of the aging muscle towards exercise induced skeletal muscle damage, and to a slower repair and adaptation response. This vulnerability can be explained by a lower level in high energy phosphates and glycogen, a slower recovery rate after exercise, and an increased accumulation of toxic metabolites within the muscle [30]. For these reasons, overtraining symptoms must be tracked, and could, if it is present, explain the absence of benefit of a prehabilitation program.

The final statement of the umbrella review is limited by the single results of the included reviews. Therefore, some outcome parameters cannot be finalized in general for some significance values are missing. Moreover, the heterogeneity of the prehabilitation programs make it difficult to draw a clear conclusion. A further subgroup would also be limited by the number of reviews which contain comparable programme parameters. At least, some studies are overlapping in the single systematic reviews and therefore are based on the same analyses (Table 4). The heterogeneity is also given concerning the quality of the single reviews, measured with the AMSTAR-2 tool. The overall quality of the presented evidence could be enhanced by strengthening the inclusion criteria. However, this would lead to an exclusion of relevant and important records. The umbrella review itself is limited in terms of language and time. Furthermore, it is not possible to discuss the long-term effects of rehabilitation, as the individual reviews included in this review do not make any statements in this regard. For reasons of survival, long-term functional independence and quality of life, it is desirable to investigate this time aspect in future studies. At least, an analysis of the cost-effectiveness of prehabilitation would complete the overall evaluation of this therapy strategy.

These results emphasize the need of developing prehabilitation programs for the aging population, tailored to the patient according to his or her baseline functional status and then adjusted in agreement with his or her tolerance to the prehabilitation program. On this basis a special program was developed: the Prehabilitation & Rehabilitation in Oncogeriatrics - Adaptation to deconditioning risk and accompaniment of Patients' Trajectories (PROADAPT), a geriatric multiprofessional intervention program, especially for older patients with cancer [31].

In summary, this umbrella review shows that prehabilitation, particularly in elderly patients with oncological diagnoses, is an effective approach to improve vital signs and overall functional status before and after necessary surgery.
